# Supplementary material for: Vitamin D Modified DSS-Induced Colitis in Mice via STING Signaling Pathway
Source: Biology (Basel). 2025 Jun 18;14(6):715. doi: 10.3390/biology14060715 (PMC12190092; doi:10.3390/biology14060715)
Supplement: Supplementary file 1 [file biology-14-00715-s001.zip › Supplementary Materials S1: 16s-report of gut microbiota/Groups/diff_analysis/RF/Phylum_Groups.RFimportance.pdf]

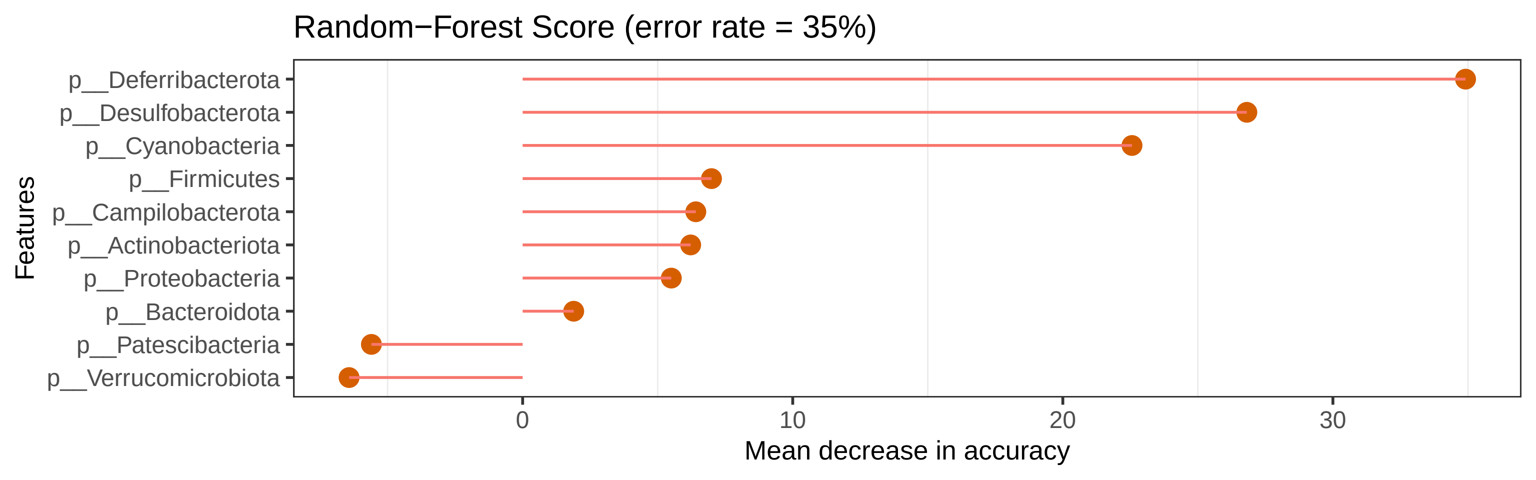

Random−Forest Score (error rate = 35%)
p__Deferribacterota
p__Desulfobacterota
p__Cyanobacteria
p__Firmicutes
p__Campilobacterota
p__Actinobacteriota
p__Proteobacteria
p__Bacteroidota
p__Patescibacteria
p__Verrucomicrobiota
Features
0
10
20
30
Mean decrease in accuracy
